# Synthetic biology and biomass conversion: a match made in heaven?

**DOI:** 10.1098/rsif.2008.0527.focus

**Published:** 2009-05-19

**Authors:** Christopher E. French

**Affiliations:** School of Biological Sciences, University of Edinburgh, Darwin Building, King's Buildings, Edinburgh EH9 3JR, UK

**Keywords:** synthetic biology, BioBricks, biomass, cellulose, ethanol, biofuels

## Abstract

To move our economy onto a sustainable basis, it is essential that we find a replacement for fossil carbon as a source of liquid fuels and chemical industry feedstocks. Lignocellulosic biomass, available in enormous quantities, is the only feasible replacement. Many micro-organisms are capable of rapid and efficient degradation of biomass, employing a battery of specialized enzymes, but do not produce useful products. Attempts to transfer biomass-degrading capability to industrially useful organisms by heterologous expression of one or a few biomass-degrading enzymes have met with limited success. It seems probable that an effective biomass-degradation system requires the synergistic action of a large number of enzymes, the individual and collective actions of which are poorly understood. By offering the ability to combine any number of transgenes in a modular, combinatorial way, synthetic biology offers a new approach to elucidating the synergistic action of combinations of biomass-degrading enzymes *in vivo* and may ultimately lead to a transferable biomass-degradation system. Also, synthetic biology offers the potential for assembly of novel product-formation pathways, as well as mechanisms for increased solvent tolerance. Thus, synthetic biology may finally lead to cheap and effective processes for conversion of biomass to useful products.

## 1. Introduction

Owing to the twin factors of declining oil reserves and increasing concern about rising carbon dioxide levels, our society must urgently seek a replacement for fossil fuels. The enormous quantities of coal, oil and gas that are used for the generation of electricity may ultimately be partially or completely replaced by other sources such as nuclear, solar-electric, solar-thermal, hydroelectric, geothermal, tidal, wave and ocean-thermal power systems. Considerable attention has also been directed to the development of electric vehicles, which may replace petrol-driven cars for short journeys. However, barring enormous improvements in battery technology, it seems unlikely that liquid fuels can be replaced in the near future for long-distance road transport, sea transport or, most particularly, aviation, which are essential for the efficient global movement of goods and people, and thus critical to the functioning of the modern world. Also, quite apart from energy uses, around 6–10 per cent of fossil fuels recovered are currently directed to the petrochemical industry (Waltz 2008), leading to a wide range of products essential to our way of life. Alternative sources of suitable molecules for these applications are urgently required. Plant-derived material is the only feasible renewable source.

A great deal of work has gone into the development of biofuels (Antoni *et al*. 2007; Dale 2008). The majority of effort is associated with bioethanol and biodiesel. Ethanol for biofuel use is produced on a large scale by fermentation using the yeast *Saccharomyces cerevisiae*. The major producers of bioethanol are Brazil and the USA. In Brazil, ethanol is derived from sucrose in sugarcane; in the USA, it is produced mainly from glucose syrup derived from maize (corn) starch, a procedure that is generally held to be less environmentally favourable than the sugarcane-based process owing to the high-energy inputs involved (Goldemberg 2007; Granda *et al*. 2007). Biodiesel is produced by esterification of fatty acids derived from vegetable oils (Hill *et al*. 2006; Granda *et al*. 2007). It has been suggested that the CO_2_ emissions generated by land clearance for growing biofuel crops may outweigh the reduction in emissions associated with reduced use of fossil fuels (Fargione *et al*. 2008; Gallagher *et al*. 2008; Searchinger *et al*. 2008). It also seems clear that production of biofuels from maize and vegetable oils is creating unacceptable competition with food, both by diverting food-grade materials and by absorbing land that could be used for growing food crops. This is considered to be one factor in the major rises in staple food prices seen in early 2008, leading to food riots in many parts of the world (Sachs 2008), though the extent to which biofuel production competes with food production has been questioned (e.g. Dale 2008). Nevertheless, it seems clear that there is limited scope for increased production of biofuels by these methods. This has led to great interest in ‘second generation’ biofuels, derived from non-food materials, particularly lignocellulosic biomass, the non-edible parts of plants (Lynd *et al*. 2005; Chang 2007; Kumar *et al*. 2008; Wackett 2008; Yuan *et al*. 2008). Potential sources of such material include agricultural and timber industry waste, waste paper and purpose-grown plant material derived from rapidly growing non-food plants such as scrub willow, switchgrass and *Miscanthus*, which can be grown on land unsuited to food production (Heaton *et al*. 2008). Since all plants consist largely of this material, in principle any plant material could be used, including mixed grasses harvested sustainably from prairie-style systems, thus avoiding the environmental issues associated with intensive monocultures.

Processes must therefore be developed for the conversion of lignocellulosic biomass into useful products. The Ideal Biofuel Producing Micro-organism (IBPM) must possess a number of independent characteristics:

- it must be able to hydrolyse cellulosic material effectively, with minimal requirement for pre-processing;
- it must be able to convert the sugars released into molecules useful as liquid fuels and/or chemical industry feedstocks;
- it must be able to produce these molecules at a high concentration without poisoning itself, in order to minimize downstream processing costs; and
- it must be capable of rapid growth in a bioreactor and suitable in other respects for use in an industrial context.

Naturally occurring micro-organisms do not fulfil these criteria. Here we will consider how the emerging discipline of synthetic biology might be applied to develop a new organism, which can be applied in such a process.

**Figure 1.**
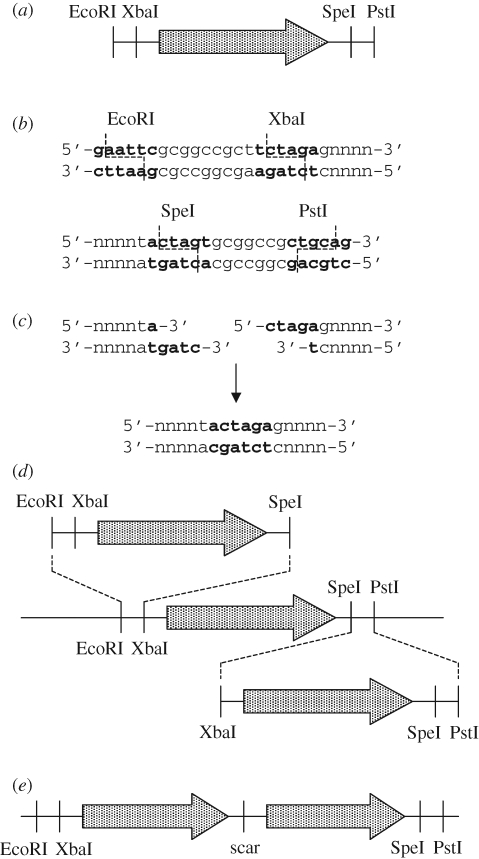
The BioBrick 1.0 assembly standard (Knight 2003; Registry of Standard Biological Parts 2009). (*a*) Each BioBrick is a length of DNA bearing a genetic component such as an open reading frame, ribosome-binding site, promoter, transcription termination sequence or any combination of these. Each BioBrick possesses EcoRI and XbaI restriction sites at the 5′ end, and SpeI and PstI sites at the 3′ end. (*b*) Standard prefix and suffix sequences for BioBricks. The six-base pair recognition sites for each restriction endonuclease are shown in bold and dashed lines indicate the staggered cuts made by each enzyme. (*c*) Ligation of an SpeI-cut end to an XbaI-cut end generates a six-base pair ‘scar’, which is not recognized by either XbaI or SpeI. (*d*) By appropriate choice of restriction enzymes, any BioBrick can be inserted either upstream or downstream of any other BioBrick. (*e*) In either case, the product, bearing both components, is also a BioBrick, bearing the same four restriction sites as the original component BioBricks. It can thus be added either upstream or downstream of any other BioBrick. In this way large and complex constructs can be built up quickly and easily from a library of standard parts.

Synthetic biology aims to construct novel biological systems from smaller components. This programme is exemplified in the concept of BioBricks (Knight 2003; Registry of Standard Biological Parts 2009): modular, interchangeable DNA components, which, due to the use of a combination of restriction sites generating compatible and incompatible sticky ends, can be assembled in any order and in any desired number to generate complex multi-gene systems, which can be further combined to any desired degree (figure 1). The original BioBrick standard is now under revision (BioBricks Foundation; www.biobricks.org), but any successor standards will certainly maintain the key principles of modularity and interchangeability. Ultimately, the decreasing cost of synthetic DNA should further simplify the assembly of complex multi-gene systems. The unprecedented flexibility offered by BioBricks and other synthetic biology approaches has tremendous potential for allowing the generation of new micro-organisms combining useful phenotypes from different sources. It is this technology that may finally allow us to generate the IBPM and make conversion of biomass to fuels and chemical feedstocks an economically competitive process.

In synthetic biology, the characteristic of robust growth in a bioreactor context is ensured by choice of an appropriate host or ‘chassis’ organism, such as *Escherichia coli*, *Bacillus subtilis* or *S. cerevisiae*, which provides background processes to support the new pathways to be introduced. The problem then becomes one of generating the new biological modules: biomass degradation, product formation and solvent tolerance. In the remainder of this paper, we will consider how synthetic biology can contribute to each of these aspects and, finally, can help to bring them all together in one organism, the IBPM.

## 2. Biomass degradation

### 2.1. The problem of biomass degradation

Of all the characteristics required for the IBPM, biomass degradation is perhaps that which has absorbed the greatest amount of research. While a great deal has been learned about the nature of microbial biomass degradation systems, efforts to transfer such systems to heterologous hosts have not been completely successful, though some progress has been made (Lynd *et al*. 2002, 2005; Kumar *et al*. 2008). Biomass degradation presents unique difficulties, which synthetic biology seems well placed to solve.

### 2.2. Enzymes and pathways of biomass degradation

Biomass consists mainly of long cellulose fibres embedded in an amorphous matrix of hemicellulose and lignin (Glazer & Nikaido 1995; Reid 1997; Lynd *et al*. 2002). The cellulose fibres consist of long chains (of the order of thousands of residues) of β-(1,4)-linked d-glucose residues, with alternating orientation so that the repeating unit is cellobiose (glucose-β-1,4-glucopyranoside). Cellulose chains are tightly packed together with insoluble hydrogen-bonded crystalline regions alternating with amorphous regions. This provides a high degree of tensile strength. Hemicelluloses are shorter molecules consisting of a mixture of monomers including d-xylose, l-arabinose, d-mannose and d-galactose, as well as sugar derivatives such as 4-*O*-methyl-d-glucuronic acid. Lignin is a complex three-dimensional polymer formed by polymerization of aromatic monomers and forms a matrix around the fibres. In principle, cellulose can be hydrolysed to release d-glucose, a suitable carbon source for the growth of micro-organisms, which could be used to produce biofuels and chemical feedstocks. However, enzymic or non-enzymic hydrolysis of cellulosic material to its component sugars is rendered difficult by the insoluble nature of the crystalline regions and the presence of lignin. Even following pre-treatment to disrupt lignin, cellulose hydrolysis is difficult and expensive, meaning that, despite its abundance, cellulose is not an economically competitive source of glucose compared to starch. Hemicellulose can be hydrolysed relatively easily, by either enzymic or non-enzymic processes, but its monomer sugars are not efficiently used by most industrially useful organisms. Lignin degradation is a more complex issue. Generally speaking, lignin is a problem in recovery of sugars from cellulosic material, and chemical and/or mechanical pre-treatment is required to disrupt lignin before hydrolysis. Another application of genetic modification and synthetic biology, which will not be discussed in this report, concerns the development of plants with reduced lignin content or altered lignin structure (Weng *et al*. 2008). Interestingly, Sarath *et al*. (2008) have pointed out that, in the case of forage crops, such as sorghum and switchgrass, varieties have already been bred with altered lignin content for increased digestability by animals, and these may also be useful in biofuel production.

Despite these issues, some bacteria and fungi are capable of rapid and efficient degradation of cellulose (Lynd *et al*. 2002). The ability to degrade amorphous cellulose, soluble cellulose analogues, such as carboxymethyl cellulose, and hemicellulose-like substrates is relatively widespread, but the ability to degrade crystalline cellulose is relatively uncommon and seems to be mainly restricted to specialized cellulose-degrading micro-organisms (Coughlan & Mayer 1992). In view of the high abundance and recalcitrance of cellulose, it is perhaps not surprising that certain micro-organisms should come to specialize in its degradation, as it offers an abundant source of carbon for which there is relatively little competition. Among cultured bacteria, efficient degradation of crystalline cellulose is mainly associated with the anaerobic spore-forming low-GC Gram-positive bacteria of the genus *Clostridium*, the aerobic high-GC Gram-positive bacteria *Cellulomonas* and some of the streptomycetes, the anaerobic Gram-negative bacteria of the *Fibrobacter* group, the aerobic Gram-negative gliding bacteria *Cytophaga* and some of the myxobacteria, and a few other groups. Fungal cellulose degraders occur in both the ascomycetes (soft rot fungi) and basidiomycetes (white and brown rot fungi). Lignin degradation has been best studied in the white rot fungi such as *Phanaerochaete chrysosporium* (Glazer & Nikaido 1995).

**Figure 2.**
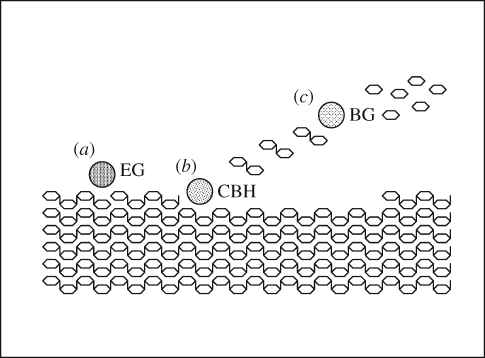
Paradigmatic process of cellulose degradation (Lynd *et al*. 2002). (*a*) Endoglucanases (EG) cleave cellulose chains at random positions to expose reducing and non-reducing ends. (*b*) Exoglucanases (cellobiohydrolases, CBH) move along the chain cleaving cellobiose residues from either the reducing or non-reducing end. (*c*) β-Glucosidases (BG) hydrolyse cellobiose to glucose, preventing accumulation of cellobiose that would inhibit cellobiohydrolase activity.

Cellulose degradation systems have been studied in a variety of organisms including certain fungi (especially *Trichoderma*, the source of the majority of commercial cellulase preparations), the aerobic bacterium *Cellulomonas fimi* and the anaerobic bacterium *Clostridium thermocellum*, as well as close relatives of these organisms. This has led to a paradigmatic view of cellulose degradation (figure 2), in which cellulose fibres are initially attacked by endoglucanases, which break random bonds in the chains to reveal free reducing and non-reducing ends. These are then attacked by exoglucanases, which move processively along the chain from either the reducing or non-reducing end, releasing cellobiose, or, in some cases, glucose or cellotetraose; exoglucanases that release cellobiose are also known as cellobiohydrolases. Cellobiose is then hydrolysed by β-glucosidases, avoiding a buildup of cellobiose, which would lead to inhibition of exoglucanases. All of these processes must operate simultaneously for effective cellulose degradation. Multiple families of enzymes with each of these classes of activity are known (Lynd *et al*. 2002; Cantarel *et al.* 2009). Exceptions to this paradigm are also known; for example, processive endoglucanases possess both endoglucanase and exoglucanase activity, cleaving chains at internal sites and then attacking the exposed ends, and, in some fungi, depolymerization of cellulose seems to occur at least partially by oxidative rather than hydrolytic processes (Baldrian & Valaskova 2008). In aerobic micro-organisms such as *Trichoderma* and *Cellulomonas*, cellulolytic enzymes are typically secreted into the growth medium (although often a considerable proportion of activity is cell associated), but in anaerobic organisms such as *Clostridium*, the enzymes are typically associated with the cell wall in a complex known as the cellulosome (Doi & Kosugi 2004; Ding *et al*. 2008), consisting of multiple interchangeable enzymic subunits attached via binding domains to a scaffolding protein that is attached to the cell wall. In addition to cellulases of these three classes, cellulose-degrading organisms invariably possess enzymes for the degradation of associated polysaccharides such as hemicellulose. This is seen even in organisms such as *Cytophaga hutchinsonii*, which are unable to assimilate sugars derived from hemicellulose hydrolysis (Xie *et al*. 2007); presumably hemicellulose breakdown is required to expose cellulose fibres.

It is noteworthy that effective cellulose-degrading organisms always seem to possess multiple enzymes of each of the classes mentioned. For example, *Trichoderma* is known to produce at least five endoglucanases, two exoglucanases and two β-glucosidases (Lynd *et al*. 2002), and *C. fimi* produces at least six endoglucanases and two exoglucanases, as well as multiple genes involved in hemicellulose degradation (Stoll 2001). In the *C. thermocellum* cellulosome, the 22 known interchangeable enzymic subunits available for incorporation include at least nine endoglucanases, four exoglucanases and five hemicellulases (Lynd *et al*. 2002; Kumar *et al*. 2008); a preliminary analysis of the draft genome sequence suggested the presence of 71 cellulosomal components, 23 of which were putative cellulases (Zverlov *et al*. 2005); and the CAZY website lists 69 glycoside hdyrolases and 4 polysaccharide lyase genes in this genome (Cantarel *et al.* 2009). Other cellulolytic organisms are less well studied, but an increasing number of genome sequences are available (Rubin 2008) and examination of some of these suggests that the same pattern holds; for example, the genome sequence of the gliding bacterium *C. hutchinsonii* (Xie *et al*. 2007) includes 11 genes annotated as possible or probable endoglucanases and four β-glucosidases, as well as numerous miscellaneous β-glycosidases, and 11 xylanases and acetylxylanesterases, which may be involved in hemicellulose degradation; oddly, no recognizable exoglucanases are present, suggesting that this organism may rely on processive endoglucanases or may use an unusual method of cellulose degradation. The pattern also holds in multi-cellular organisms; for example, the genome sequence of the plant-parasitic nematode *Meloidogyne incognita* was reported to encode 21 cellulases and 6 xylanases, in addition to multiple other enzymes potentially involved in plant cell wall degradation (Abad *et al*. 2008).

The question arises as to why multiple representatives of each class of enzyme are beneficial. Presumably this is owing to the complexity of the substrate—while each individual β-1,4-glycosidic bond may be chemically identical, the complex nature of the surrounding material means that they are not in identical contexts. One might therefore expect to observe synergy in cellulose degradation between different enzymes of the same class, as well as between enzymes of different classes. Synergy between cellulases of different classes has been well studied (Lynd *et al*. 2002; Zhang & Lynd 2004), but relatively few studies seem to describe synergy between representatives of the same class. One example concerns the endoglucanases CelZ and CelY of the plant pathogenic bacterium *Erwinia chrysanthemi*; these were reported to display synergy in degradation of amorphous cellulose, with CelY having preference for cleavage of longer chains, whereas CelZ showed greater activity with shorter chain substrates, which were not effectively cleaved by CelY (Zhou & Ingram 2000, 2001). Clearly, the role of intra-class synergy in biomass degradation requires further investigation. With regard to studies of synergy between enzymes of different classes, synergy is observed in some cases, but not in others; indeed, Andersen *et al*. (2008) recently reported that a mixture of endoglucanase Cel45A and exoglucanase Cel6A of *Humicola insolens* and β-glucosidase of *Penicillium brasilianum* showed synergy on amorphous cellulose, but on crystalline cellulose the enzymes actually seemed to inhibit each other, an effect attributed to competition for binding sites on the cellulose. Other studies with different enzymes have reported the opposite effect, with synergy seen on crystalline cellulose but not amorphous cellulose (Valjamae *et al*. 1999; Zhang & Lynd 2004). Concerning synergy between larger groups of enzymes, an interesting recent report (Zverlov *et al.* 2008) describes mutant strains of *C. thermocellum* with disruptions of the scaffoldin gene *cipA*, encoding the protein that normally keeps all of the various enzymic components of the cellulosome in close spatial proximity to each other and to cellulose-binding components. The mutant strains showed a similar level of activity against soluble substrates, but activity against crystalline cellulose was reduced about 15-fold compared with the wild type, indicating a strong effect of proximity. Clearly, synergy is a complex issue; quantitative kinetic models (Zhang & Lynd 2004, 2006) may help to clarify the situation.

Although the gene sequences of many known and presumed cellulases are now available, considerable research effort is being directed towards the search for new cellulase genes from unculturable organisms by metagenomic techniques; for example, Warnecke *et al*. (2007) reported a metagenomic study of the micro-organisms of the termite hindgut, a highly active cellulolytic micro-environment, and other studies in progress are cited by Rubin (2008). However, in such densely populated systems, with many interacting species, the question of synergy between cellulases, particularly between those produced by different organisms, will become even more complex.

### 2.3. Commercial biomass conversion processes

Cellulose conversion processes have mainly focused on ethanol production. The basic process involves the use of fungal cellulases, produced mainly by *Trichoderma reesei*, to partially hydrolyse cellulose (pre-treated to disrupt lignin), and then fermentation of the resulting glucose in a conventional process using *S. cerevisiae* (Glazer & Nikaido 1995; Lynd *et al*. 2002). An extension to this process is simultaneous saccharification and fermentation (SSF), in which cellulases are added to a bioreactor with cellulose and the production organism, so that cellulose degradation is concurrent with glucose assimilation and product formation. This removes the issue of product inhibition of cellulose degradation and reduces the complexity of the process. In simultaneous saccharification and cofermentation, pentose sugars released by hydrolysis of hemicellulose are fermented in the same process. This requires use of an organism able to ferment these sugars, which are not used by wild-type *S. cerevisiae* strains. Strains of *S. cerevisiae* able to ferment xylose have been developed by expression of xylose isomerase of *Piromyces*, or xylose reductase and xylitol dehydrogenase of *Pichia* (e.g. Ho *et al*. 1998; Hector *et al*. 2008; Hughes *et al*. 2009; as well as many other reports) or by natural selection of non-recombinant *S. cerevisiae* to improve xylose fermentation (Attfield & Bell 2006). Also, strains of *E. coli* and its close relative *Klebsiella oxytoca*, which can naturally use xylose and arabinose, have been engineered to produce ethanol with high yields (discussed further below). The logical final development is ‘consolidated bioprocessing’ (CBP; Lynd *et al*. 2002, 2005), in which a single organism produces cellulases, assimilates the sugars released and produces the desired product. Despite recent decreases in the cost of exogenous fungal cellulases used in current processes, the calculations of Lynd *et al*. (2005) indicate that CBP will still lead to a substantial decrease in overall production costs. Unfortunately, no known natural organism possesses the necessary combination of characteristics. One potential approach is to improve ethanol formation by natural cellulolytic organisms such as *C. thermocellum* and *C. phytofermentans*; the other is to transfer a cellulose degradation system into a robust industrial organism capable of high-level biofuel production.

A number of companies have been established with the production of cellulosic ethanol as their main aim, and a great deal of public and venture capital funding has been obtained. Several companies, including Bioethanol Japan, Mascoma, Iogen, Verenium and POET, are reportedly operating pilot scale plants, with more expected to open in the near future.

### 2.4. Transfer of cellulose degradation capability to heterologous hosts

Many reports have described the expression of cellulase genes in heterologous hosts, both for research purposes and with the explicit aim of endowing the recipient with the ability to degrade biomass effectively (Lynd *et al*. 2002, 2005). To summarize, it appears that the expression of highly active enzymes has in many cases been problematic and, while several reports have described recombinant organisms capable of degrading amorphous cellulose and assimilating the resulting sugars, effective degradation of crystalline cellulose without the addition of exogenous cellulases remains elusive. Here, we will consider only a few recent reports giving an indication of the current state of the art.

Many reports describe expression of fungal cellulases in *S. cerevisiae*, with the aim of producing a yeast strain capable of directly fermenting cellulose to ethanol. For example, Cho *et al*. (1999) reported the integration of multiple copies of the bifunctional endo/exoglucanase of *Bacillus* sp. DO4 and the β-glucosidase of *Bacillus circulans* into the chromosomal DNA of *S. cerevisiae*. Enzyme activity was detected, and there appeared to be somewhat enhanced growth in the presence of cello-oligosaccharides, but it was concluded that higher expression levels would be required to allow effective growth and ethanol production. Cho & Yoo (1999) later reported that the use of this strain in an SSF process significantly reduced the requirement for addition of commercial cellulases. Fujita *et al*. (2004) reported generation of a recombinant strain of *S. cerevisiae* expressing *T. reesei* endoglucanase 11 and cellobiohydrolase 11 together with β-glucosidase 1 of *Aspergillus aculeatus*. The three enzymes were expressed as fusion proteins attached to the cell surface. The recombinant strain was able to produce about 3 g l^−1^ ethanol from amorphous cellulose, but growth with cellulose as sole carbon source was not reported; the organism was pre-grown on a rich medium before being provided with cellulose. Den Haan *et al*. (2007*a*) recently described the generation of a strain of *S. cerevisiae* co-expressing endoglucanase EG1 of the filamentous fungus *T. reesei* and β-glucosidase BGL1 of the yeast *Saccharomycopsis fibuligera*, and demonstrated that the recombinant strain could grow with amorphous cellulose (phosphoric acid swollen cellulose, PASC) as sole carbohydrate source, and produce ethanol, though, for reasons not entirely clear, only 1 g l^−1^ ethanol was produced. The authors stated that this was the first report of a yeast strain able to grow with cellulose as sole carbohydrate source, though it should be noted that the growth medium also contained yeast extract and peptone. More effective cellulose degradation will require the expression of an exoglucanase (cellobiohydrolase); den Haan *et al*. (2007*b*) investigated the expression of four cellobiohydrolase genes from the fungi *T. reesei*, *Aspergillus niger* and *P. chrysosporium* in *S. cerevisiae* and reported that all four were successfully expressed and secreted, though at rather low levels.

Where bacteria, rather than yeasts, are used as the host, most reports concern the introduction of cellulase genes into strains of *E. coli* or its close relative, *K. oxytoca*, expressing the *pet* (production of ethanol) operon (Ingram *et al*. 1987), which allows these organisms to generate ethanol with high yield (discussed further in §3 below). *Klebsiella oxytoca* is similar to *E. coli* but has a greater ability to assimilate and degrade cello-oligosaccharides and xylo-oligosaccharides. The most advanced of these cases seems to be the report of *E. coli* and *K. oxytoca* strains expressing the endoglucanases CelY and CelZ from the plant pathogenic bacterium *E. chrysanthemi* (Zhou *et al.* 2001). This organism was able to ferment amorphous cellulose to ethanol without the addition of exogenous cellulases and was also reported to require smaller amounts of supplemental cellulases when fermenting crystalline cellulose in an SSF process (Zhou & Ingram 2001).

Thus, despite many experiments expressing various combinations of cellulases in different hosts, effective growth with crystalline cellulose as sole carbon source remains elusive. One obvious issue is expression level. Owing to the rather low specific activities of cellulases, relatively large quantities of cellulases must be produced and secreted in order for sugars to be released at a high enough rate to support growth. Lynd *et al*. (2005) have calculated that the required expression levels are within achievable ranges, but suggest that these levels have not yet been achieved with active cellulases. Another potential issue is the relatively low energy yield of the homoethanologenic fermentation, even compared with other fermentations such as the mixed acid fermentation; coupled with the high energy requirement for cellulase production and secretion, this may make energy balance a significant issue for effective ethanol production from cellulosic material. The allocation of ATP between growth and cellulase production may be an important parameter for manipulation (van Walsum & Lynd 1998).

### 2.5. Role for synthetic biology

It seems clear that a number of issues must be resolved in order to generate the cellulose-degradation module required for the IBPM. The simplest interpretation of the failure to achieve efficient cellulose degradation in a heterologous host is that cellulase expression levels are simply not sufficient (Lynd *et al*. 2005). Given the technologies available for high-level protein expression and secretion in both yeast and bacteria in the biopharmaceuticals industry, this would seem to be a relatively straightforward problem to address. Synthetic biology using modular components allows the easy combination of coding sequences with libraries of promoters, enhancers and secretion signals, to determine empirically which systems provide the best results. Perhaps a more interesting possibility is that synergy, both within and between the different classes of cellulase, plays a more critical role than has been assumed. This might explain why cellulose-degrading micro-organisms, in contrast to, for example, starch-degrading organisms, always seem to contain a battery of different enzymes with very similar predicted activities. Production of a proper blend of perhaps a dozen or so enzymes might result in a much higher level of activity, reducing the amount of total protein that must be secreted, and thus the energy cost associated with enzyme production and secretion. Without extremely detailed characterization of the properties of all of these enzymes, it will be very difficult to predict exactly which blends of enzymes are likely to give the best results. One way forward would be a massive parallel screening exercise in which a large library of different combinations of cellulase-encoding genes was prepared and screened in parallel for release of sugars from various different classes of cellulosic substrate, allowing empirical determination of the best systems. The modular, interchangeable nature of components such as BioBricks is ideally suited to such experiments (figure 1). A library of putative biomass-degrading enzymes from different organisms could be generated, and parallel assembly processes used to generate organisms expressing a variety of different combinations, which could then be screened for effective biomass degradation ability. In this way, one could hope to determine empirically which combinations of enzymes are most effective for degradation of different classes of biomass substrate. The results obtained from such an exercise could then be used to generate hypotheses that could be used to guide further experiments, ultimately resulting in an efficient set of heterologous cellulose-degradation genes suitable for incorporation into the IBPM, or perhaps several alternative sets suited to different types of substrate.

We have made a small start in this direction, generating BioBricks based on the major endoglucanases and exoglucanases of *C. fimi* and the putative endoglucanases of *C. hutchinsonii* (Elfick *et al*. 2008; S. Lakhundi, C.-K. Liu and C. E. French, unpublished data). Considering the ongoing rapid development of synthetic biology and the urgency of this problem, no doubt others are following similar approaches.

## 3. Product formation

### 3.1. Biofuel products

Following conversion of biomass to assimilable sugars, the IBPM must convert this material to a product that can be used as a liquid fuel and/or chemical feedstock. Ethanol has attracted the most attention, but other products have also been considered, including *n*-butanol, other higher alcohols, fatty acid ethyl esters, liquid terpenoids and alkanes (Antoni *et al*. 2007; Atsumi & Liao 2008; Fortman *et al*. 2008; Wackett 2008). Here, we will briefly consider some of the leading contenders, with particular reference to the prospect of transferring the required production pathway into a new chassis.

### 3.2. Ethanol

The potential product that has attracted the most attention is ethanol. Many organisms produce small amounts of ethanol during anaerobic growth, but production of ethanol as the main or sole product (homoethanologenic fermentation) seems to be rather rare, perhaps because of the low energy yield of this fermentation, and has mainly been studied in the yeast *S. cerevisiae* and the bacterium *Zymomonas mobilis*. Formation of ethanol from pyruvate (a universal intermediate of glucose assimilation) is straightforward, requiring only two enzymes: pyruvate decarboxylase (PDC), which converts pyruvate to acetaldehyde and CO_2_; and alcohol dehydrogenase (ADH), which reduces acetaldehyde to ethanol, re-oxidizing NADH. Thus, transfer of these two genes to a host organism, perhaps with diversion of pyruvate away from other pathways, should be sufficient to allow ethanol production. Early experiments in this area were performed by Ingram *et al*. (1987), who used the *pdc* and *adh* genes of *Z. mobilis* to generate an artificial operon designated *pet* (production of ethanol), which was successfully expressed in *E. coli*, as well as the related organism *K. oxytoca* (Ohta *et al*. 1991), which has a greater capacity to take up cello-oligosaccharides and xylo-oligosaccharides (Burchhardt & Ingram 1992; Wood & Ingram 1992). This technology is reportedly being applied by Verenium and Bioethanol Japan. The *pet* operon was reported to work poorly in Gram-positive bacteria; an alternative system, using *pdc* and *adh* from the Gram-positive bacteria *Sarcina ventriculi* and *Geobacillus stearothermophilus*, respectively, has been reported to work well in Gram-positive hosts such as *Bacillus megaterium* (Talarico *et al*. 2005). To assuage any possible concern about the use of genetically modified bacteria on a large scale, Kim *et al*. (2007) reported the generation of a non-recombinant strain of *E. coli*, which ferments glucose and xylose to ethanol, despite lacking PDC. This involved inactivating mutations in the genes encoding pyruvate-formate lyase and lactate dehydrogenase, which are required for the major fermentation pathways in wild-type *E. coli*, and altered regulation of pyruvate dehydrogenase to allow its expression under fermentative conditions. Production of acetyl-CoA via pyruvate dehydrogenase rather than pyruvate-formate lyase leads to excess NADH production, meaning that a higher proportion of acetyl-CoA must be reduced to ethanol rather than converted to acetate as in the standard mixed acid fermentation.

### 3.3. Other alcohols and ketones

Ethanol has a number of advantages as a biofuel: because of its increasing use in the USA and Brazil, a growing infrastructure exists for its distribution, and vehicles able to use it, either in pure form or blended with gasoline, are increasingly available. Blends such as E10 (10% ethanol, 90% gasoline) can be used in unmodified engines, but higher levels of ethanol require engine modifications. ‘Flex-fuel’ cars, able to run on either gasoline or E85 (85% ethanol, 15% gasoline) are increasingly popular. However, as a fuel, ethanol also has a number of disadvantages. Most importantly, recovery by distillation requires a large energy input, and high water miscibility means that it cannot be transported in pipelines, but must be delivered in tankers. It is also rather corrosive. Therefore, a number of other microbial fuel products are under consideration. Perhaps the best developed of these is *n*-butanol (Antoni *et al*. 2007). Butanol can reportedly be used directly in standard petrol engines without modification; it has a higher energy density than ethanol, is less corrosive and is not fully miscible with water, limiting its tendency to absorb water from the surroundings. Butanol is produced, along with acetone and ethanol, in the ‘ABE’ fermentation, using *Clostridium acetobutylicum* (Lee *et al*. 2008). Historically, this was an important process, but became generally uneconomical compared with petrochemical processes around the 1960s. With increasing oil costs, and increasing concern about CO_2_ emissions, there has been a resurgence of interest in fermentative butanol production. The *C. acetobutylicum* process suffers from relatively low yields and low product concentrations, owing to the toxicity of butanol to the producing organism. Inui *et al*. (2008) and Atsumi *et al*. (2008*a*) have both reported expression of the butanol production pathway in *E. coli*, leading to production of significant quantities of *n*-butanol under aerobic and anaerobic conditions. Another of the ABE pathway products, acetone, is also a potentially interesting product, and has been produced in aerobically grown *E. coli* expressing four *C. acetobutylicum* genes (Bermejo *et al*. 1998); due to its high volatility, acetone could be recovered directly from the exhaust gas stream. Co-expression of a secondary alcohol dehydrogenase allows acetone produced in this way to be reduced to isopropanol, another potential biofuel or feedstock (Hanai *et al.* 2007; Jojima *et al*. 2008). Non-fermentative processes for the production of four- and five-carbon alcohols have also been explored. For example, Atsumi *et al*. (2008*b*) recently reported a process for the production of branched chain alcohols, such as isobutanol, in *E. coli*, by diverting and reducing 2-ketoacid intermediates from amino acid biosynthetic pathways.

### 3.4. Other potential biofuels

A microbial process for the production of biodiesel, dubbed ‘micro-diesel’, has also been reported (Kalscheuer *et al*. 2006). This process used *E. coli* expressing the *pet* operon, leading to ethanol production, together with an acyltransferase from *Acinetobacter baylyi*, capable of reacting ethanol with fatty acids to generate fatty acid ethyl esters, which can be used as biodiesel. Exogenous fatty acids had to be added, but the authors speculated that upregulation of the fatty acid biosynthesis pathway might allow direct production of biodiesel from sugars. The fatty acid biosynthetic pathway can also be redirected to the production of long-chain alcohols and fatty esters, which are also potential biofuels under commercial investigation (Zhihou & Fernando 2008).

From a practical point of view, perhaps the simplest replacement for fossil hydrocarbons would be non-fossil hydrocarbons. The bacterium *Vibrio furnissii* has been reported to produce substantial quantities of *n*-alkanes (Park *et al*. 2001, 2005), apparently via reduction of long-chain alcohols (Park 2005); however, another recent study found no evidence for hydrocarbon production in this organism (Wackett *et al*. 2007), leaving this promising pathway in limbo, although at least one patent has been filed suggesting that long-chain fatty alcohols can be enzymically reduced to alkanes (Zhihou & Fernando 2008). Certain algae produce high levels of hydrocarbons, including terpenoids and alkanes (Raja *et al*. 2008). Perhaps the best studied of these is *Botryococcus braunii* (Metzger & Largeau 2005), which has been reported to produce high levels (up to 60% of dry weight) of both long-chain alkenes and branched-chain isoprenoid hydrocarbons. Considerable research has gone into the development of processes for the large-scale growth of such algae for hydrocarbon production (Chisti 2007), with a number of companies established specifically for this purpose, but to date, very little genetic work appears to have been done with such algae, meaning that transfer of the hydrocarbon synthesis pathway to a more convenient host would not be possible without a great deal more research. Given the level of interest in this area and the decreasing costs of sequencing, *B. braunii* would seem to be a strong candidate for a genome sequencing project.

Another possible source of liquid fuels is the isoprenoid biosynthetic pathway. Isoprenoids are a hugely diverse family of molecules synthesized from five-carbon precursors (isopentenyl pyrophosphate and dimethylallyl pyrophosphate) via the mevalonate pathway, in yeast and other eukaryotes, or the non-mevalonate (DXP) pathway, in plastids and most bacteria. Many reports have described metabolic engineering of isoprenoid biosynthetic pathways for the production of a wide variety of products (Kirby & Keasling 2008). For example, Keasling *et al*. have reported the upregulation of isoprenoid synthesis in both *E. coli* (Martin *et al*. 2003) and *S. cerevisiae* (Ro *et al*. 2006) for the production of the antimalarial drug artemisinin. This process is being further developed by Amyris Biotechnologies, which is also investigating the production of liquid substitutes for gasoline and diesel produced by enzymic or chemical reduction of 5- and 15-carbon products from the terpenoid biosynthetic pathway (Withers *et al*. 2007; Renninger & McPhee 2008; Renninger *et al*. 2008*a*,*b*).

Of course, there are many other useful products that could be produced from cellulose apart from biofuels. One particularly intriguing possibility would be the conversion of cellulose to starch (Elfick *et al*. 2008). Standard synthetic biology chassis organisms such as *E. coli*, *B. subtilis* and *S. cerevisiae* produce glycogen as a carbon storage material. Glycogen is an α-1,4- and α-1,6-linked glucose polymer substantially similar to the precursor of starch in plants. While the processes leading to starch production are not entirely understood, mutational studies have implicated a set of enzymes, including isoamylase and granule-bound starch synthase, which are believed to play the major roles (Ball & Morell 2003), and a synthetic approach might be the ideal way to elucidate this pathway. A moderately efficient process for conversion of cellulosic biomass to starch could revolutionize agriculture, allowing the cultivation of plants chosen for rapid growth rather than those optimized for diversion of photosynthate to starch production. Starch thus produced could be used as an animal feed, currently a major use for the US grain harvest (Dale 2008), or as an intermediate in biofuel production, since hydrolysis of starch is much more rapid and inexpensive than hydrolysis of cellulosic biomass. This could increase the amount of grain available for human food use.

### 3.5. Potential roles for synthetic biology

The assembly of complex metabolic pathways is one of the flagship applications for synthetic biology. The modular nature of BioBricks and similar parts facilitates the construction of large multi-gene constructs using standardized promoters, ribosome-binding sites and termination sequences to control expression levels, as well as allowing genes to be rearranged easily, new genes to be added and other manipulations to be performed using a set of standard techniques. The remarkable results achieved by teams of undergraduate students in the annual International Genetically Engineered Machine competition (iGEM 2008) hosted by the Massachusetts Institute of Technology testify to the simplicity of assembling metabolic pathways using BioBricks. Wider adoption of this approach can only make it faster and easier to generate new product-formation modules for incorporation into the IBPM.

## 4. Solvent tolerance

### 4.1. The importance of solvent tolerance

Biofuel products must form a combustible liquid around room temperature. Such hydrophobic substances are invariably more or less harmful to cells, primarily through damage to membranes. For economical purification, it is important that the products be produced at a high concentration, but this is generally limited by the ability of the cells to survive such exposure. It is therefore of great interest to examine the mechanisms by which cells survive exposure to damaging levels of solvents and the means by which this tolerance can be enhanced.

### 4.2. Solvent tolerance in bacteria

Solvent tolerance in various organisms has been linked to a wide variety of mechanisms, including alteration of *cis*:*trans* membrane lipid ratios, alteration of membrane lipid headgroups, alteration to lipopolysaccharide in the outer membrane of Gram-negative bacteria, production of efflux pumps that push hydrophobic molecules out of the cell and release of hydrophobic molecules in membrane vesicles (Ramos *et al*. 2002; Sardessai & Bhosle 2002). These studies have mainly concerned solvent-susceptible mutants and suppressors of such mutations. Relatively few studies seem to have reported increases in solvent tolerance by overexpression of particular proteins. In the case of *E. coli*, most solvent-tolerant mutants were found to overexpress the regulatory gene *marA*, leading to overexpression of a number of other genes; solvent tolerance seemed to be particularly associated with the efflux pump formed by AcrAB together with the outer membrane protein TolC, since deletion of any of these three genes abolished the effect (Aono 1998). More recent studies have focused on analysis of genes with altered expression patterns in mutants with increased solvent tolerance. One such study led to the discovery that solvent tolerance could be enhanced by overexpression of *glpC*, encoding the membrane anchor subunit of the anaerobic glycerol-3-phosphate dehydrogenase GlpABC (Shimizu *et al*. 2005). This effect seemed to be independent of any catalytic activity of this enzyme, suggesting that it was in fact because of alteration of the membrane properties owing to insertion of excess GlpC. In a similar study, overexpression of the mannose phosphotransferase uptake transporter ManXYZ was found to increase organic solvent tolerance (Okochi *et al*. 2007*a*). In this case, overexpression of the individual subunits was not effective, suggesting that the intact transporter was required. Overexpression of *manXYZ* was also found to increase the solvent tolerance of a *marA*-overexpressing strain, indicating an additive or synergistic effect. In a final example, it was found that overexpression of the chaperone prefoldin from *Pyrococcus horikoshii* led to increased solvent tolerance (Okochi *et al*. 2007*b*). In contrast to the preceding examples, this did not seem to be associated with altered cell surface properties as such, but rather with decreased protein denaturation.

### 4.3. Solvent tolerance in yeast

Relatively few studies have reported increased solvent tolerance in eukaryotes. Recent reports mainly concern the mutant *S. cerevisiae* strain KK-211, isolated on the basis of iso-octane tolerance (Kawamoto *et al*. 2001). The mutation was later identified as being a point mutation in a transcription factor, Pdr1p (Matsui *et al*. 2008), which affected the transcription of multiple genes (Miura *et al*. 2000; Matsui *et al*. 2006). The mechanistic basis of the enhanced solvent tolerance does not seem to have been established. However, an interesting alternative approach was reported by Zou *et al*. (2002), who expressed a library of random fragments derived from cDNA as cell surface-exposed fusion proteins. One clone, designated n13, was found to show enhanced tolerance to *n*-nonane; the DNA insert was found to encode 91 amino acids from ORF YGR193C. While the basis of the enhanced solvent tolerance is not altogether clear, it is presumably related to altered cell surface properties, and this may be a good method for empirical generation of solvent-tolerant strains.

### 4.4. Role for synthetic biology

In view of the multiple mechanisms that have been demonstrated to lead to enhanced solvent tolerance, it would be very interesting to determine whether solvent tolerance could be further enhanced by combining these mechanisms (Okochi *et al*. 2007*a*). As discussed above, the use of synthetic biology tools such as BioBricks would provide a simple and effective way to examine the co-expression of different combinations of factors, casting light on their interactions. Libraries of BioBricks encoding genes associated with different solvent tolerance mechanisms could easily be generated and tested in different combinations, screening directly for survival in the presence of different solvents. This approach could rapidly provide a set of solvent tolerance modules suited for different products, which could be combined with different product-formation modules for incorporation into the IBPM.

## 5. Conclusions

Economical conversion of cheap, abundant, renewable biomass to valuable products will require the combination of a range of different characteristics that do not naturally occur in any one organism. We have seen that metabolic pathways for product formation can be transferred from one host to another, and that synthetic biology offers improved methods for investigating synergy between the many gene products involved in biomass degradation and solvent tolerance. Synthetic biology also offers a suite of rapidly improving tools for the combination of multiple genetic modules in a single chassis (host) organism. For example, different combinations of biomass degradation genes could be generated to provide a set of biomass-degradation modules suited to different types of cellulosic substrate materials. Similarly, product-formation modules could be generated allowing the production of different products suitable for use as biofuels or chemical industry feedstocks, and these could be combined with solvent-tolerance modules suitable for protecting the organism against each particular product. For any desired combination of substrate and product, one could simply choose the appropriate modules for substrate degradation, product formation and product tolerance, and combine them in a robust, high performance chassis to generate an IBPM for each application. Thus, the fortuitous combination of the rapidly developing discipline of synthetic biology and the increasingly urgent requirement for improved biomass conversion processes offers the potential for a sustainable, oil-free future.
